# Supplementary material for: Unveiling the Bioactive Potential of the Invasive Jellyfish Phyllorhiza punctata Through Integrative Transcriptomic and Proteomic Analyses
Source: Biomolecules. 2025 Aug 4;15(8):1121. doi: 10.3390/biom15081121 (PMC12383608; doi:10.3390/biom15081121)
Supplement: Supplementary file 1 [file biomolecules-15-01121-s001.zip › Suplementary Material/FileS3/PEPFOLD4/XP_065059926_12011/clusters.html]

 PEP-FOLD clusters


Download archive of all models  here   

|  |  |  |
| --- | --- | --- |
| # | file | sOPEP |
| # cluster 1 - Size: 21 |  |  |
|  | XP\_065059926\_12011-model1 | -36.1995 |
|  | XP\_065059926\_12011-model1.1 | -36.0627 |
|  | XP\_065059926\_12011-model1.2 | -35.9865 |
|  | XP\_065059926\_12011-model1.3 | -35.9865 |
|  | XP\_065059926\_12011-model1.4 | -35.9833 |
|  | XP\_065059926\_12011-model1.5 | -35.9485 |
|  | XP\_065059926\_12011-model1.6 | -35.9169 |
|  | XP\_065059926\_12011-model1.7 | -35.9056 |
|  | XP\_065059926\_12011-model1.8 | -35.8834 |
|  | XP\_065059926\_12011-model1.9 | -35.6765 |
|  | XP\_065059926\_12011-model1.10 | -35.5403 |
|  | XP\_065059926\_12011-model1.11 | -35.4628 |
|  | XP\_065059926\_12011-model1.12 | -35.4565 |
|  | XP\_065059926\_12011-model1.13 | -35.4271 |
|  | XP\_065059926\_12011-model1.14 | -35.4091 |
|  | XP\_065059926\_12011-model1.15 | -35.3945 |
|  | XP\_065059926\_12011-model1.16 | -35.3281 |
|  | XP\_065059926\_12011-model1.17 | -35.2805 |
|  | XP\_065059926\_12011-model1.18 | -35.1991 |
|  | XP\_065059926\_12011-model1.19 | -35.1521 |
|  | XP\_065059926\_12011-model1.20 | -33.8351 |
| # cluster 2 - Size: 48 |  |  |
|  | XP\_065059926\_12011-model2 | -33.7339 |
|  | XP\_065059926\_12011-model2.1 | -33.6561 |
|  | XP\_065059926\_12011-model2.2 | -33.6293 |
|  | XP\_065059926\_12011-model2.3 | -33.5687 |
|  | XP\_065059926\_12011-model2.4 | -33.5586 |
|  | XP\_065059926\_12011-model2.5 | -33.5402 |
|  | XP\_065059926\_12011-model2.6 | -33.5379 |
|  | XP\_065059926\_12011-model2.7 | -33.4836 |
|  | XP\_065059926\_12011-model2.8 | -33.4457 |
|  | XP\_065059926\_12011-model2.9 | -33.4398 |
|  | XP\_065059926\_12011-model2.10 | -33.3705 |
|  | XP\_065059926\_12011-model2.11 | -33.3535 |
|  | XP\_065059926\_12011-model2.12 | -33.3379 |
|  | XP\_065059926\_12011-model2.13 | -33.2836 |
|  | XP\_065059926\_12011-model2.14 | -33.2814 |
|  | XP\_065059926\_12011-model2.15 | -33.2788 |
|  | XP\_065059926\_12011-model2.16 | -33.2727 |
|  | XP\_065059926\_12011-model2.17 | -33.2687 |
|  | XP\_065059926\_12011-model2.18 | -33.239 |
|  | XP\_065059926\_12011-model2.19 | -33.2209 |
|  | XP\_065059926\_12011-model2.20 | -33.2174 |
|  | XP\_065059926\_12011-model2.21 | -33.1996 |
|  | XP\_065059926\_12011-model2.22 | -33.1942 |
|  | XP\_065059926\_12011-model2.23 | -33.1826 |
|  | XP\_065059926\_12011-model2.24 | -33.176 |
|  | XP\_065059926\_12011-model2.25 | -33.1728 |
|  | XP\_065059926\_12011-model2.26 | -33.1694 |
|  | XP\_065059926\_12011-model2.27 | -33.1505 |
|  | XP\_065059926\_12011-model2.28 | -33.1475 |
|  | XP\_065059926\_12011-model2.29 | -33.1437 |
|  | XP\_065059926\_12011-model2.30 | -33.1427 |
|  | XP\_065059926\_12011-model2.31 | -33.1396 |
|  | XP\_065059926\_12011-model2.32 | -33.1366 |
|  | XP\_065059926\_12011-model2.33 | -33.1183 |
|  | XP\_065059926\_12011-model2.34 | -33.0339 |
|  | XP\_065059926\_12011-model2.35 | -33.0308 |
|  | XP\_065059926\_12011-model2.36 | -33.009 |
|  | XP\_065059926\_12011-model2.37 | -33.0069 |
|  | XP\_065059926\_12011-model2.38 | -33.0012 |
|  | XP\_065059926\_12011-model2.39 | -32.9874 |
|  | XP\_065059926\_12011-model2.40 | -32.9687 |
|  | XP\_065059926\_12011-model2.41 | -32.9206 |
|  | XP\_065059926\_12011-model2.42 | -32.8439 |
|  | XP\_065059926\_12011-model2.43 | -32.8328 |
|  | XP\_065059926\_12011-model2.44 | -32.7656 |
|  | XP\_065059926\_12011-model2.45 | -32.7028 |
|  | XP\_065059926\_12011-model2.46 | -32.6793 |
|  | XP\_065059926\_12011-model2.47 | -32.5237 |
| # cluster 3 - Size: 31 |  |  |
|  | XP\_065059926\_12011-model3 | -33.6977 |
|  | XP\_065059926\_12011-model3.1 | -33.6057 |
|  | XP\_065059926\_12011-model3.2 | -33.586 |
|  | XP\_065059926\_12011-model3.3 | -33.5626 |
|  | XP\_065059926\_12011-model3.4 | -33.5171 |
|  | XP\_065059926\_12011-model3.5 | -33.4857 |
|  | XP\_065059926\_12011-model3.6 | -33.4652 |
|  | XP\_065059926\_12011-model3.7 | -33.4461 |
|  | XP\_065059926\_12011-model3.8 | -33.4391 |
|  | XP\_065059926\_12011-model3.9 | -33.4017 |
|  | XP\_065059926\_12011-model3.10 | -33.398 |
|  | XP\_065059926\_12011-model3.11 | -33.3675 |
|  | XP\_065059926\_12011-model3.12 | -33.3575 |
|  | XP\_065059926\_12011-model3.13 | -33.3495 |
|  | XP\_065059926\_12011-model3.14 | -33.3492 |
|  | XP\_065059926\_12011-model3.15 | -33.3079 |
|  | XP\_065059926\_12011-model3.16 | -33.303 |
|  | XP\_065059926\_12011-model3.17 | -33.298 |
|  | XP\_065059926\_12011-model3.18 | -33.2975 |
|  | XP\_065059926\_12011-model3.19 | -33.258 |
|  | XP\_065059926\_12011-model3.20 | -33.2497 |
|  | XP\_065059926\_12011-model3.21 | -33.2334 |
|  | XP\_065059926\_12011-model3.22 | -33.1852 |
|  | XP\_065059926\_12011-model3.23 | -33.1597 |
|  | XP\_065059926\_12011-model3.24 | -33.1486 |
|  | XP\_065059926\_12011-model3.25 | -33.1179 |
|  | XP\_065059926\_12011-model3.26 | -33.0271 |
|  | XP\_065059926\_12011-model3.27 | -32.9883 |
|  | XP\_065059926\_12011-model3.28 | -32.9103 |
|  | XP\_065059926\_12011-model3.29 | -32.9062 |
|  | XP\_065059926\_12011-model3.30 | -32.8398 |
|  |  |  |
